# On the Present State of Vaccination in Oxford

**Published:** 1816-02

**Authors:** Richard Walker


*For the London Medical and Physical Journal.*

### *On the present State of Vaccination in Oxford;* by Richard Walker, Esq.

IN your Journals of October and November last, you did me the favour of inserting some fresh observations on Vaccination, collected chiefly in the month of May last, at which time the natural small-pox began to make its appearance in Oxford, in some instances where there was good reason to suppose previously that such persons were secured by having passed through vaccination in its most perfect form, as well as in several others who had passed through vaccination in a less satisfactory manner.

From

From that time to the date of the present communication, the natural small-pox, (which had been almost entirely unknown here for several years past, viz. since inoculation has been abandoned, and vaccination almost generally substituted in its stead,) has been more prevalent and general, and fatal indeed, than even before vaccination was introduced and inoculation practised. I believe I am justified in stating that such has been the case in the vicinity of Oxford; the credit of vaccination, which, until this time, had preserved the entire confidence of the superior classes, and amongst the lower classes of people had few dissentients latterly; has now, especially amongst the latter, received such a check, or rather shock, that it will be extremely difficult, if possible, ever to restore its shaken credit amongst them: but even amongst these, it seems to be understood and believed, that future small-pox is rendered milder by previous vaccination; but this is not sufficient for them, and nothing seems now to satisfy them but inoculation, this being known to afford absolute and permanent security.

This failure of security from vaccination, has been confined almost exclusively to the lower order of people; the reason of which is obvious: first, in consequence of its being imperfectly or less carefully performed, and its progress not so duly watched as in the superior classes; and, secondly, because this class of persons are more exposed to its contagion in consequence of their more promiscuous intercourse with the affected, and other aiding circumstances; and, moreover, it is a well-known fact now, that vaccinated persons, although they resist the infection at one time, are yet susceptible of it at another time; hence in no instance whatever is there absolute permanent security.

The appearance of persons strongly marked in the face by the effect of recent small-pox is now, although by no means common, not very strange to us — one now and then presenting themselves, which is a novelty we have long been unaccustomed to.

Having given a faithful account, I trust, of the present state of vaccination, which unfortunately is far from being a favourable one, I would readily, were it in my power, proceed to point out a remedy for this defect.

Were vaccination conducted according to the test of its perfection, and the cautions recommended in my last paper, adhered to, although I am convinced there would not, in all instances, afford absolute security; yet, I am still of opinion, that thus managed, vaccination would ever preserve a sufficient degree of credit, to supersede inoculation.

I have,

I have, however, myself witnessed lately anomalies in vaccination, to which I was formerly a stranger, and which I am apt now to suspect arises from a real deterioration in the virus of cow-pock matter in passing through an almost endless series of the human system; for instance, the pustule which I have described in my last paper as local, appears to be far more common than formerly, and is probably the consequence of a deteriorated or less active state of the fluid used for propagating the affection.

It is no uncommon circumstance now to meet with persons under vaccination, having not only two punctures, but three, and even four; this I have seen myself. In many instances, the vaccinated pustules, notwithstanding this precaution, have passed through their progress in a puny state, and without the least efflorescent inflammation, the *sine qua non*, as I consider it, of the complete progress of the pustule. In one person I have seen four pustules, the effect of four punctures in one arm; these were accompanied with a considerable degree of inflammation, as might be expected, but not the *genueris areola*, and not the slightest indisposition; hence, even here there is no reasonable prospect of security. This reminds me, if I might be allowed to digress thus, of the speech of Macbeth, when the ghost of murdered Banquo at a banquet takes the seat, apparently in *propria persona*, allotted to Macbeth himself, to this purport—"The times have been when one mortal blow would lay a man at rest; but now, with twenty mortal wounds, the business yet remains unfinished." In short, I think now, the subject or merits respecting inoculation and vaccination, considering all circumstances, which I have mentioned before and need not repeat here, may be briefly summed up thus, omitting all minor considerations, viz. that inoculation is the only absolute security against future small-pox;* and that vaccination is not equally certain, even when passed through in the most perfect manner. Secondly, the test or criterion of perfect inoculation is so apparent, viz. the variolous eruption, however slight, (preceded by an evident indisposition,) the *sine qua non*, as I consider it, of absolute security, that no one can overlook it; wherever, in vaccination, the indisposition which has been reckoned the *sine qua non* of perfection in this affection, is confessedly by all practitioners frequently wanting, or so slight as to pass unnoticed. Thirdly, the fluid in

* The circumstance of the natural small-pox following inoculation is a case barely possible to happen, and is so far removed from probability as never to occasion apprehension.

the the eruptive pustule (which is not exactly the case in the inoculated part,) whether proceeding from inoculation or natural small-pox, may be relied upon in its serous state, and likewise in its purulent state, for communicating small-pox; but in vaccination in its serous state only.* Fourthly, the fluid of small-pox undergoes no deterioration in passing through the human system, at least, it is always sufficiently active to produce small-pox; and, moreover, there is ever an opportunity of referring to the eruptive pustule, which is, in every respect, precisely of the same properties or activity, whether taken from a person inoculated, or who has received it casually or naturally; whereas, the fluid of cow-pox, there is great reason to suppose, degenerates, becoming by repeated propagation, less active and less certain in its effect; and, moreover, the means of referring occasionally to its original source, the affected cow, is extremely confined. The only circumstance I think worthy of being set against this is, that inoculation is a means of propagating natural small-pox; hence, it may be said, admitting the individual himself is benefited by it, the community at large are sufferers; to this it might be answered, that that which is beneficial to the individual would be equally so to the community at large, viz. the general practice of inoculation at an early age, either before or immediately after dentition is past.

Thus, then, the question rests or hinges; absolute and permanent security from future small-pox is to be obtained from inoculation alone, respecting the perfection or completion of which there is no difficulty in discerning: next to this is vaccination immediately from the cow, beyond which I have good reason to think there is no absolute certainty of producing the cow-pox to the human species in its most energetic or effective state.†

So

* It is a well-established fact, that the fluid of small-pox and of cow-pox are most active in their serous state; hence, it follows, that in vaccination it is then only fit for use; whereas, (as I have had frequent experience of myself,) the fluid of small-pox pustules, though weaker in a purulent state, is still fit for communicating small-pox; moreover, I have good reason to think, that the fluid of small-pox in any advanced purulent state, produces a milder kind of small-pox by inoculation, than when in a serous state.

† I know a young gentleman who was vaccinated immediately from the cow, when vaccination was first introduced; in addition to the part vaccinated going through its progress perfectly at the ordinary period, he became indisposed in as severe and similar a manner

So long as vaccination upon the present system is pursued, it is advisable in all instances, that a pustule be preserved entire and untouched throughout its progress; if it be robbed of its lymph, whilst in its serous state, it may interrupt or interfere with the complete effect of such pustule; and after this state of the lymph is past, it is universally allowed to be unfit for propagating the affection to another. Hence, a puncture in each arm becomes necessary, if it be required to transfer the affection to others, one for this latter purpose alone, or chiefly so.

In inoculation one puncture, or pustule, is sufficient: first, because it is much more acute than vaccinating fluid; and, secondly, because the virus in inoculation enters the system at an earlier stage than in vaccination,* the symptoms of absorption appearing ordinarily about the seventh or eighth day; but in vaccination, (according to my own observation)

---

manner as in sickening in consequence of inoculation, and of as nearly as long continuance, but without any subsequent eruption. This corresponds with what occurs to persons who receive cow-pox casually from the cow, as in milking, and, likewise, with little abatement, at first propagating the affection from one human subject to another; but the case is now, the virus having progressively degenerated, obviously different; it is agreed on by all practitioners, that the indisposition is frequently wanting, or so slight as scarcely to be noticed, which corresponds with my own experience, although the part vaccinated, may have made a satisfactory progress; and, moreover, that now we see the vaccinated pustule very commonly pass through its whole progress in a puny, languid, unsatisfactory manner, and propagate similar ones to others, unless there happen to be in the habit a more than ordinary degree of excitement. The remedy, and only perfect remedy, under these circumstances is obvious, but not very attainable—vaccination immediately from the cow alone.

* I have lately met with a decided or unequivocal instance, when the virus in inoculation was received into the system and produced small-pox without the least local effect being produced on the part inoculated: this person and another were inoculated on the same morning, the puncture shewed no signs of infection, but an indisposition and moderately variolous eruption ensued exactly synchronous in time with the other, and it did not appear that there were any means of the affection having been taken casually. A similar circumstance has been noticed as having happened, by some persons, but questioned by others. This, however, I consider a case removed from all doubt; in this instance, an eruptive pustule, about the time the others appeared, formed on the puncture, the puncture itself having faded or died away; this pustule was evidently an eruptive pustule differing entirely from a puncture pustule.

if they appear at all, about the eighth or ninth day. The fluid, both for inoculation and vaccination, is ordinarily taken on the eighth day, and frequently a day or two earlier.

It may be proper to mention here, that this representation of the present state of vaccination in Oxford, is to be understood as coming from myself individually, and consequently that I, alone, am responsible for its correctness.

There are two opinions, I think, which naturally present themselves respecting the cow-pox, viz. that it must either be an affection naturally incident to the cow, at least not naturally incident to the human species, or a propagation to the cow of small-pox by a milker, and returned again somewhat modified to the human species.

In either instance, it might be reasonably apprehended, that vaccination might fail in the object intended, ultimately; for, if the former of these were the fact, not being indigenous or congenial to the human system, it might in time wear itself out without occasional renovation from its original source; and, if the other were the fact, that in time it would acquire its original character of small pox.

It will be inferred, perhaps, from the tenor of this paper, that, upon the whole, I am rather an advocate for inoculation than vaccination; I acknowledge, that were inoculation universally adopted, I should be so, but not as it ever has been and ever must be expected to be, were it returned to again, practised partially; unless vaccination should hereafter be found, in consequence of its inefficiency, to be the greater evil, or the least beneficial of the two.

Amidst the various anomalies that sometimes occur, both in inoculation and vaccination, is the difference in time at which the infection commences. I have a vaccinated patient at this time, in whom there were no signs of infection at the puncture till the seventh day; after which it proceeded progressively on in the most satisfactory manner.

*Oxford; Jan.* 1, 1816.

---

Whilst we return our sincere thanks to Mr. Walker for his very candid communication, we are obliged to make a few remarks. It will be seen by our Journal, vol. xv. page 582, that, when vaccination was renewed from the cow at the Small-pox Hospital, no difference could be perceived in the human vesicle between insertion from this fresh fluid, procured under Dr. Jenner's inspection, at Berckley, and what had gone through a succession of 1000 human subjects. Respecting the origin of the disease, we cannot help regretting that a subject capable of demonstration, should be left in doubt. Why will not some village practitioner inoculate a cow, or repeat the experiments made with the grease in horses?—Edit.

